# Concurrent treatment of iliofemoral deep vein thrombosis and submassive pulmonary embolism through a single popliteal venous access

**DOI:** 10.1016/j.jvscit.2026.102530

**Published:** 2026-09-29

**Authors:** Scott S. Berman

**Affiliations:** Section of Vascular Surgery, The University of Arizona College of Medicine, Pima Heart and Vascular, Tucson, AZ

**Keywords:** Aspiration thrombectomy, Deep vein thrombosis, Popliteal vein access, Pulmonary embolism, Venous thromboembolism

## Abstract

Concomitant extensive iliofemoral deep vein thrombosis (DVT) and submassive pulmonary embolism (PE) present a therapeutic challenge for endovascular management. We report the case of a 74-year-old man presenting with extensive right iliofemoral and inferior vena cava DVT associated with bilateral PE and right ventricular strain. Using right popliteal venous access, thrombus was successfully removed from the inferior vena cava and iliofemoral venous system, and both pulmonary arteries. This case demonstrates the feasibility of treating both extensive lower-extremity DVT and submassive PE concurrently through a single popliteal venous access using one thrombectomy platform.

Venous thromboembolism (VTE), comprising deep vein thrombosis (DVT) and pulmonary embolism (PE), represents a spectrum of the same disease process and remains an important cause of cardiovascular morbidity and mortality. Although anticoagulation remains the foundation of treatment for both, selected patients with extensive proximal DVT or clinically significant PE may benefit from catheter-based thrombus removal.^1^, ^2^ For acute PE, prospective studies have demonstrated that catheter-based mechanical or aspiration thrombectomy can rapidly reduce pulmonary arterial thrombus burden and right ventricular strain with relatively low rates of major bleeding.^3^, ^4^ Similarly, percutaneous mechanical thrombectomy has emerged as a treatment option for selected patients with extensive symptomatic proximal DVT.^5^, ^6^, ^7^

Published experience with single-session treatment of both DVT and PE through a single popliteal venous access are limited. To our knowledge, there are no previous reports describing treatment of concomitant lower-extremity DVT and PE using a single popliteal venous access and computer-assisted aspiration thrombectomy platform during the same procedure. Written informed consent for publication of this case report and accompanying images were obtained from the patient.

## Case report

A 74-year-old man presented with 1 week history of worsening shortness of breath. He had no specific risk factors for VTE. His physical examination was remarkable for some mild dyspnea at rest, and he was on 4 L/min of oxygen via nasal cannula. He had mild right lower-extremity edema.

Duplex imaging demonstrated acute appearing DVT involving the right proximal popliteal vein through the femoral, common femoral, external iliac, and common iliac veins and into the inferior vena cava (IVC). This was confirmed by a computed tomographic (CT) scan with venous phase (Fig 1). A CT angiogram of the chest noted PE involving multiple branches of the right and left pulmonary arteries (Fig 2) with an elevated right ventricle to left ventricle ratio of 1.4.

**Fig 1:**
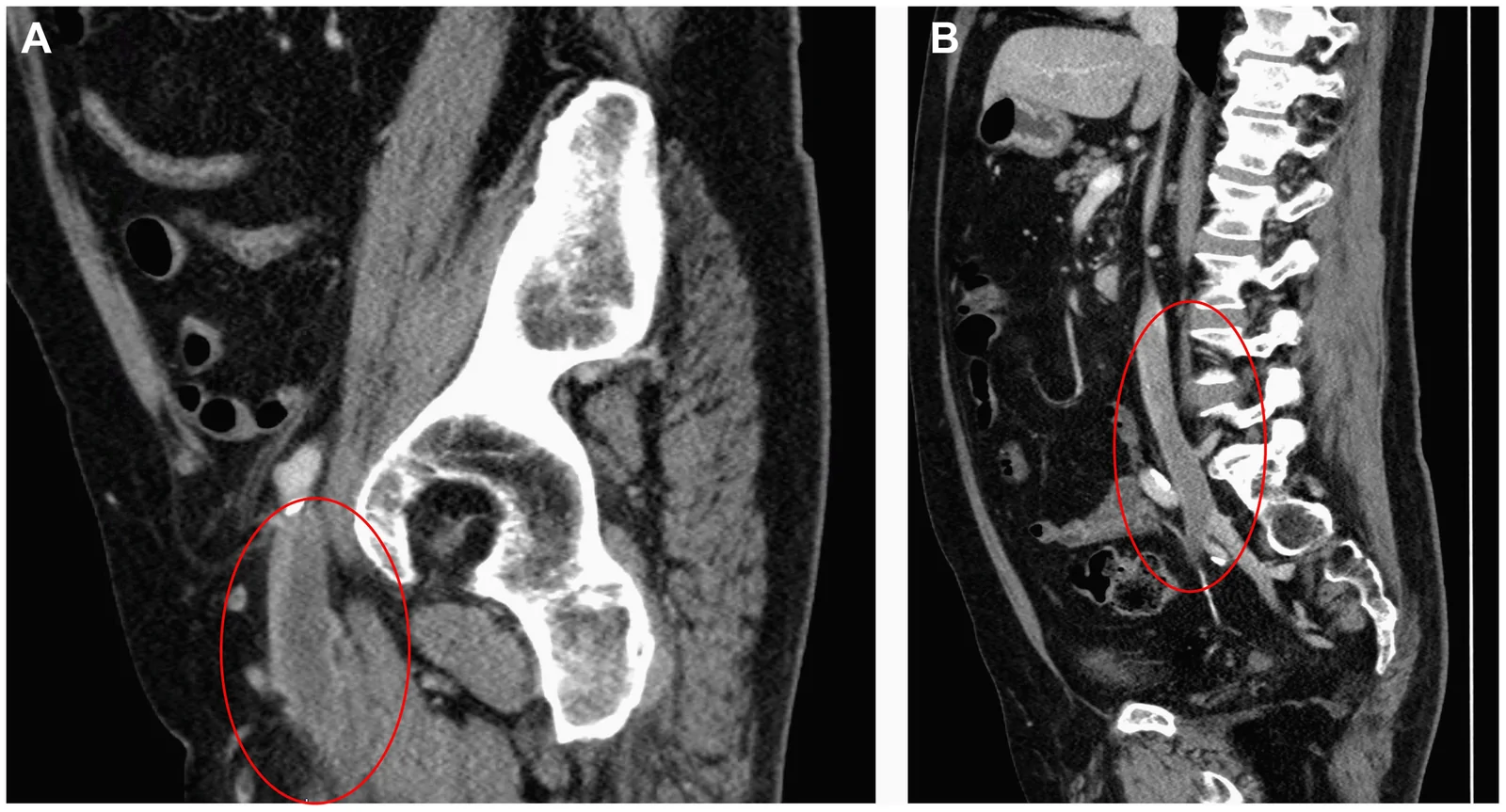
Computed tomographic scan with venous phase shows occlusion (*red circle*) of the iliofemoral veins **(A)** and inferior vena cava **(B)**.

**Fig 2:**
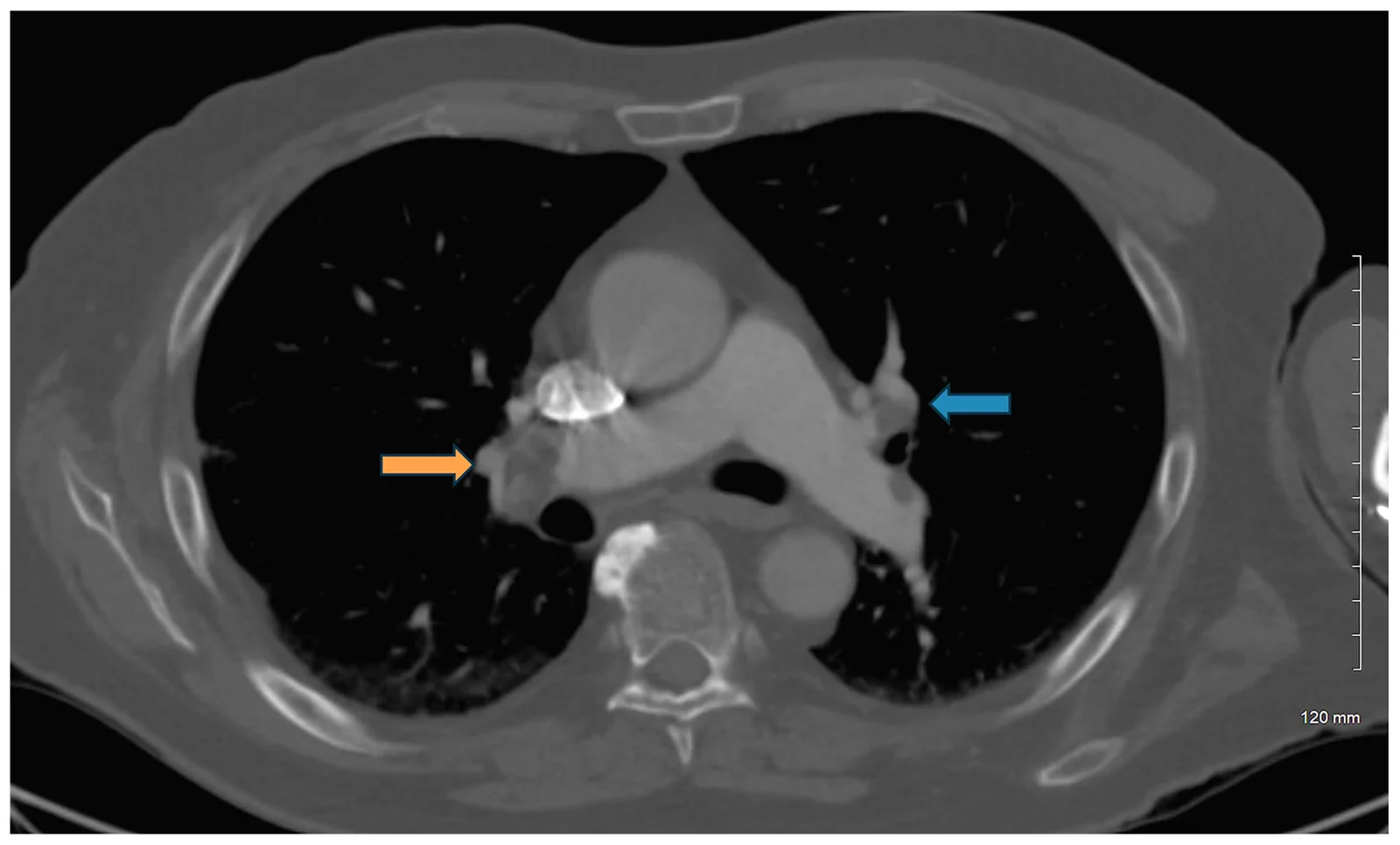
Computed tomographic angiogram reveals pulmonary embolism involving the right (*red arrow*) and left (*blue arrow*) pulmonary arteries.

Echocardiography results included a dilated right ventricle and McConnell sign. Treatment options were discussed with the patient to include anticoagulation alone vs catheter-based treatment of the DVT and PE. Catheter-based therapy was offered for the DVT and PE based upon the extent of DVT and the presence of right heart strain, respectively. The patient opted to undergo catheter-based thrombectomy of his DVT and PE.

With the patient in the prone position and receiving conscious sedation, the right popliteal vein was accessed and a 6F sheath placed. Venography demonstrated occlusion all the way from the proximal popliteal vein to the iliac vein with a tongue of thrombus in the IVC just above the iliac vein confluence (Fig 3). The Penumbra Lightning Flash 3.0 16F (Penumbra Inc) system was prepared. The device was laid out over the operative field, and it was confirmed that the device was long enough to reach into the pulmonary artery (PA) branches from the popliteal approach in this patient. A 17F Element sheath (Penumbra Inc), supplied separately, was placed in the popliteal vein, and the Penumbra Lightning Flash 3.0 system was used to successfully remove thrombus from the IVC and iliofemoral venous system, restoring patency confirmed by venography (Fig 4).

**Fig 3:**
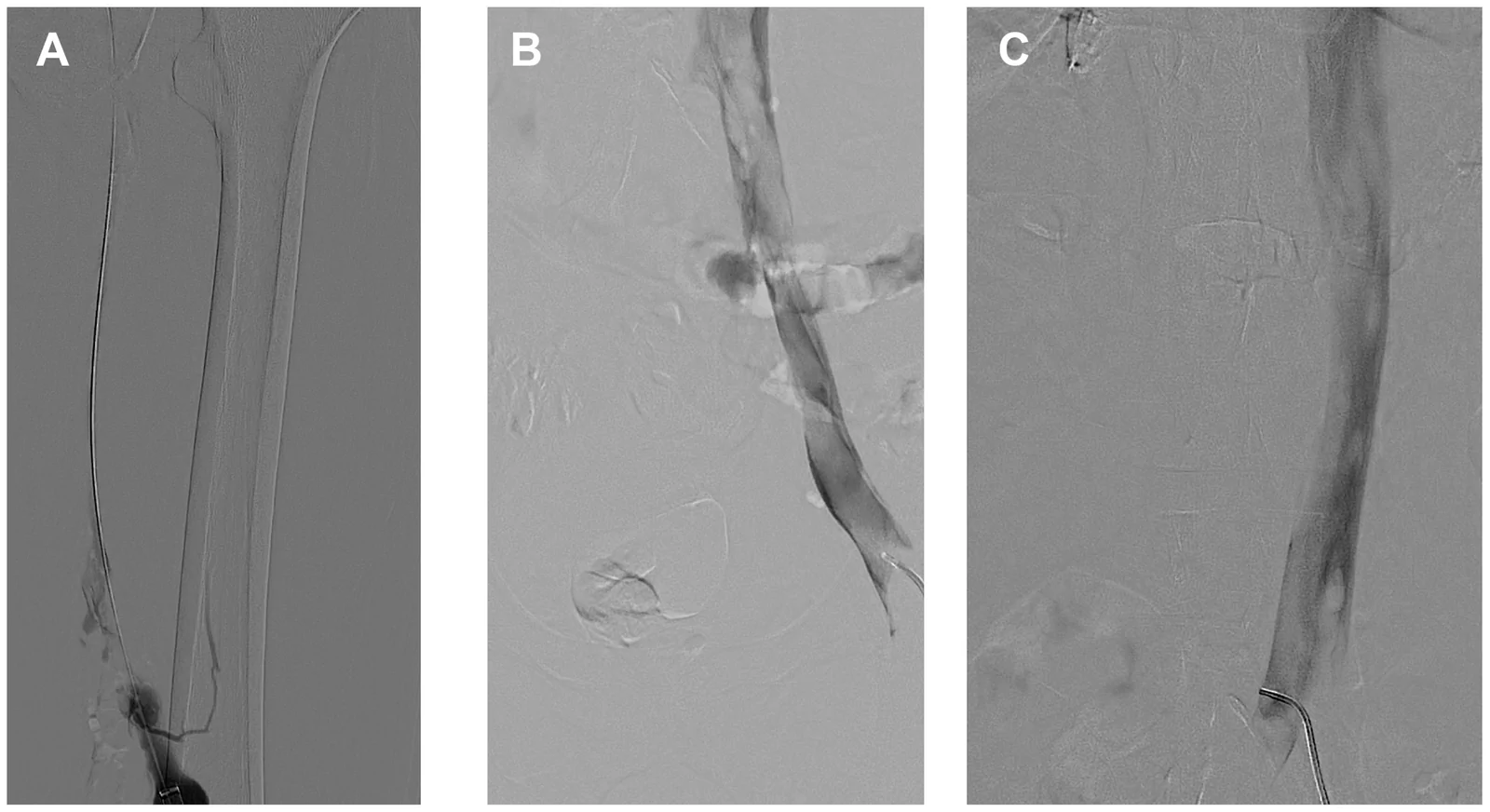
Venography demonstrates acute deep vein thrombosis involving the right femoral vein **(A)**, iliac veins **(B)** and inferior vena cava **(C)**.

**Fig 4:**
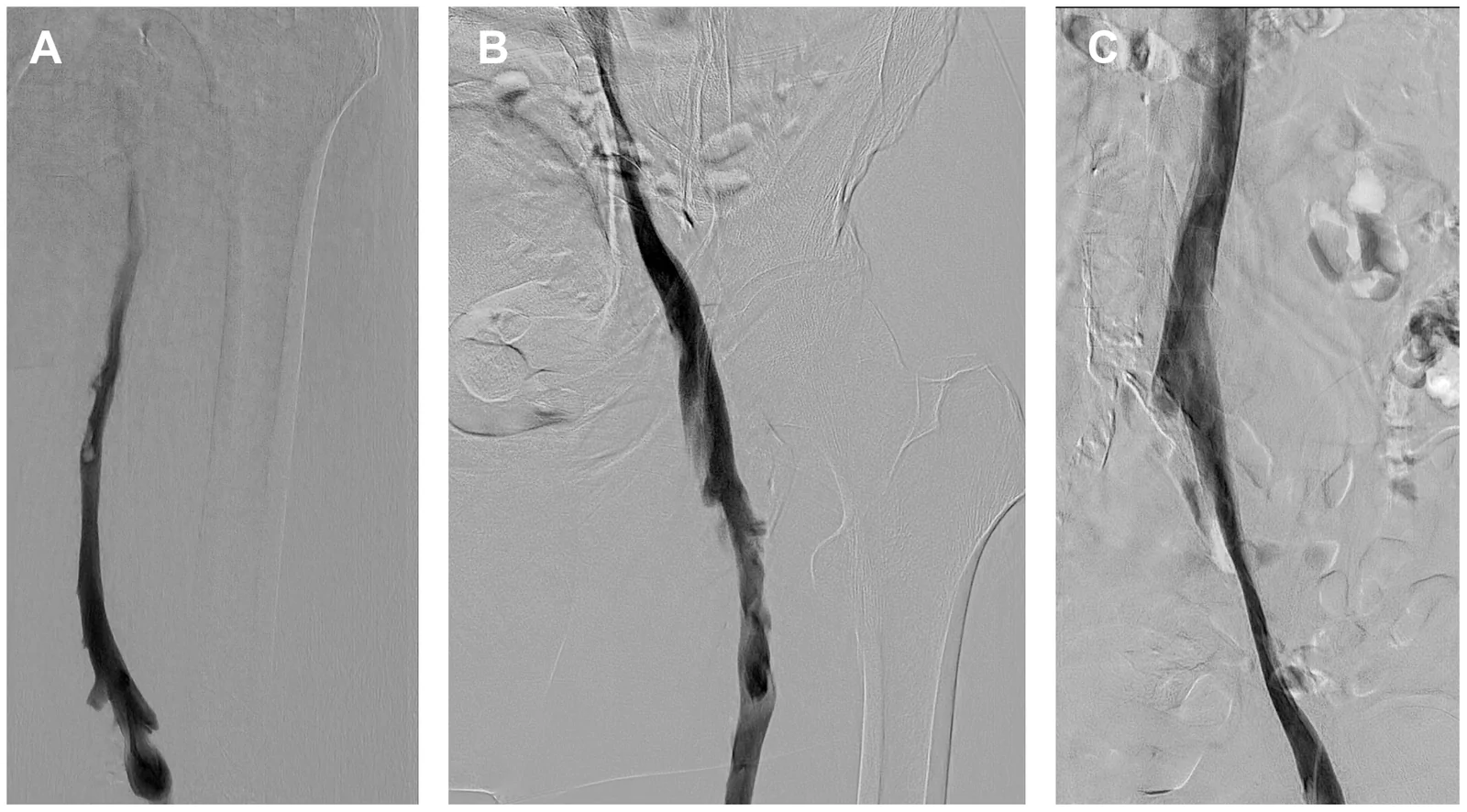
Posth thrombectomy venography demonstrates restoration of patency in the right femoral vein **(A)**, iliac veins **(B)** and inferior vena cava **(C)**.

An MIV catheter (Merit Medical) was then used to traverse from the right atrium, through the right ventricle and into the right PA. The Penumbra Lightning Flash was advanced into the right PA and successful clearing of the PE was accomplished followed by the left PA. The Penumbra was removed. Intravascular ultrasound was then performed from the IVC to the popliteal vein to confirm clearing of the clot and also to evaluate the IVC for any evidence of pathology at the right renal vein orifice that might be related to the renal mass seen on CT. The sheath was removed, and the leg wrapped. Total time of the procedure was 57 minutes, and the patient was admitted to a telemetry unit after observation in the postanesthesia care unit. Estimated blood loss collected in the Penumbra aspiration cannister was 300 mL. He was left on unfractionated intravenous heparin for 24 hours postprocedure according to our institution’s high-dose protocol. The patient did not require any transfusions during his hospitalization. During the procedure an activated clotting time of >250 seconds was verified. The clot aspirated from the IVC, iliofemoral DVT and PEs appear in Fig 5, *A* and *B*, respectively.

**Fig 5:**
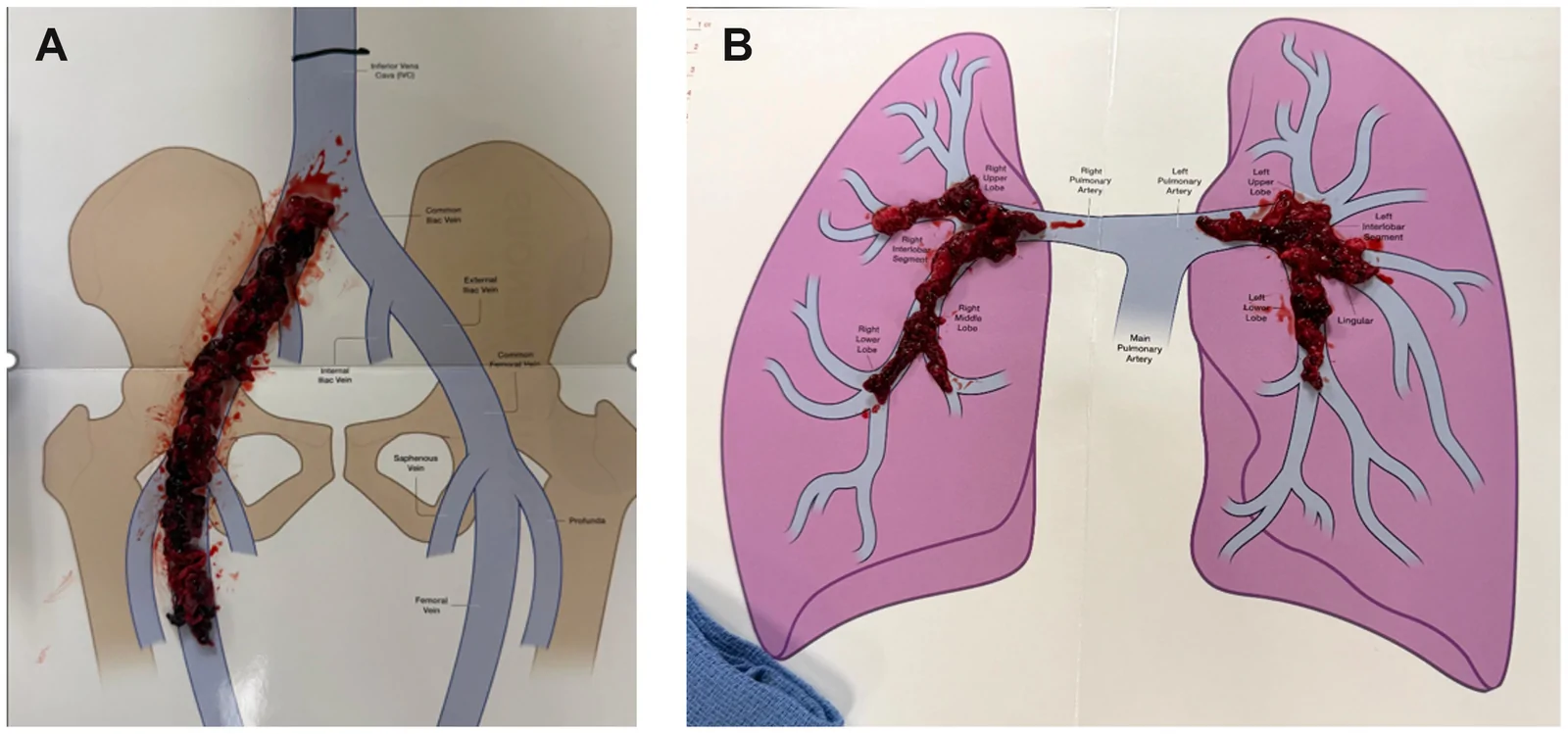
Acute appearing clot aspirated from the right femoral, iliac veins and inferior vena cava **(A)** and bilateral pulmonary arteries **(B)**.

On postprocedure day 1, the patient was comfortable on room air and had no dyspnea even with ambulation. The patient’s right leg swelling had resolved. He was converted to oral anticoagulation and discharged home on postprocedure day 2.

## Discussion

DVT and PE are anatomically and pathologically linked manifestations of VTE, yet invasive treatment has traditionally approached them as separate procedural targets. Lower-extremity thrombectomy is commonly performed through popliteal, femoral, or jugular venous access, whereas pulmonary thrombectomy is generally performed through femoral or jugular access. The present case demonstrates the feasibility of using a single popliteal venous access site to treat both extensive iliofemoral DVT and associated PE during the same procedure.

Mechanical thrombectomy has emerged as an important treatment option for selected patients with intermediate-risk PE and extensive symptomatic iliofemoral DVT. For PE, aspiration thrombectomy can rapidly reduce thrombus burden and right ventricular strain without the need for fibrinolytic therapy. The Evaluating the Safety and Effectiveness of the Indigo Aspiration System in the Treatment of Pulmonary Embolism trial demonstrated a significant reduction in right ventricular/left ventricular ratio following aspiration thrombectomy, supporting the feasibility and effectiveness of this approach.^3^ Similarly, contemporary experience with mechanical thrombectomy for iliofemoral DVT has demonstrated high rates of thrombus removal, restoration of venous flow, and symptomatic improvement in appropriately selected patients. The ClotTriever Outcomes Registry and more recent reports using computer-assisted aspiration thrombectomy systems have shown favorable technical success and safety profiles while avoiding thrombolytic exposure.^8^

The most distinctive feature of the strategy described in this report is the treatment of both manifestations of VTE through a single venous access site using the same large-bore aspiration platform. Pieraccini et al^9^ reported six patients treated simultaneously for DVT and PE with aspiration thrombectomy, demonstrating technical success for PE treatment but less consistent success in recanalizing the DVT component. Bedi et al^10^ subsequently described concomitant treatment of massive iliofemoral DVT and PE; however, separate venous access sites were required, and catheter-directed thrombolysis necessitated intensive care monitoring and prolonged infusion therapy. In contrast, the present case was completed through a single popliteal venous access using computer-assisted aspiration thrombectomy alone. According to the manufacturer, the Penumbra Lightning computer-assisted aspiration system uses a microprocessor-driven clot detection algorithm that continuously analyzes pressure and flow characteristics at the catheter tip. When thrombus is engaged, the system maintains full aspiration; when free-flowing blood is detected, it automatically modulates or interrupts suction through rapid valve control. This allows efficient thrombus extraction while reducing unnecessary blood loss and providing real-time audiovisual feedback to the operator.

Several potential advantages may be associated with this approach. A single venous puncture eliminates the need for additional femoral or jugular access. When popliteal access is already required for treatment of iliofemoral DVT, the same sheath can provide a direct route through the recanalized venous system to the right atrium and pulmonary arteries. The use of a single thrombectomy platform may also simplify procedural workflow while allowing immediate treatment of both pulmonary arterial obstruction and residual peripheral thrombus burden.

This report is limited by its single-patient design. Technical feasibility does not establish superiority over conventional approaches, and the strategy may not be suitable in patients with unfavorable anatomy, inability to traverse the iliofemoral venous system, inadequate catheter length, or conditions that increase the risks of right-heart catheter manipulation. Additional experience will be required to determine the reproducibility, safety, and clinical benefits of this technique.

## Conclusions

Single popliteal venous access may provide a feasible route for single-session computer-assisted aspiration thrombectomy of concomitant iliofemoral DVT and PE. This approach allows treatment of both manifestations of acute VTE using a single access site and thrombectomy platform. Further experience is needed to define patient selection, procedural safety, and comparative clinical benefit.

## Funding

None.

## Declaration of generative AI and AI-assisted technologies in the writing process

During the preparation of this work, the author used ChatGPT to develop a reference list for the manuscript topic and format the manuscript. After using this tool, the author reviewed and edited the content as needed and takes full responsibility for the content of the publication.

## Disclosures

None.
